# Effective treatment of human breast tumors by chimeric CCL2 and CCL8 diphtheria toxin cytotoxic peptides

**DOI:** 10.1080/15384047.2026.2688479

**Published:** 2026-07-02

**Authors:** Bernardo Chavez, Vitali Sikirzhytski, Chang-uk Lim, Ioulia Chatzistamou, Hippokratis Kiaris

**Affiliations:** a Department of Drug Discovery and Biomedical Sciences, College of Pharmacy, University of South Carolina, Columbia, SC, USA; b Department of Pathology, Microbiology and Immunology, School of Medicine, University of South Carolina, Columbia, SC, USA; c Peromyscus Genetic Stock Center, University of South Carolina, Columbia, SC, USA

**Keywords:** Tumor targeting, MCP-1, MCP-2, personalized therapy, drug uptake, PDX, resistance

## Abstract

**Objective:**

Chemokine receptors play crucial roles in tumor onset and progression, but the high redundancy between ligands and their receptors limits the possibilities to leverage them therapeutically for cancer management. To overcome this limitation, we developed chimeric chemokine peptides in which CCL2 and CCL8 were conjugated to diphtheria toxin (DT) and evaluated their antitumor activity.

**Methods:**

Cytotoxic peptides DTCCL2 and DTCCL8 were produced as recombinant proteins, and their anticancer activity was tested *in vitro* in cultured cells and *in vivo* in tumor-bearing mice. The uptake of the cytotoxic analogs was evaluated before and after therapy in tumor explants *ex vivo*.

**Results:**

Both analogs were cytotoxic to breast cancer cells in vitro and produced significant anticancer activity in vivo in mice bearing human breast cancer lines and hormone-negative breast cancer patient-derived xenografts (PDXs). In vitro, the peptide conjugates exhibited overlapping uptake profiles, with about 80% of the breast cancer cells being positive for both peptides and about 15%–20% of the cells being negative for either or both of the cytotoxic peptides. In tumor explants cultured ex vivo, simultaneous positivity for DTCCL2 and DTCCL8 increased to >95%, with less than 5% of the cells showing neither DTCCL8 nor DTCCL2 uptake. Treatment of breast cancer-bearing mice with DTCCL8 or DTCCL2 significantly inhibited tumor growth and prolonged survival in the PDX model.

**Conclusion:**

These results support the feasibility of cytotoxic peptide conjugates for breast cancer management and show that receptor expression profiles in vitro do not accurately forecast tumoral positivity.

## Introduction

Cancer remains a fatal disease affecting about 20% of the population.^1^ State-of-the-art therapeutic strategies in personalized medicine leverage the availability of patient-specific molecular lesions for cancer treatment. However, such precision medicine strategies, even according to most optimistic estimations, can help only a fraction of patients. A recent study analyzing 47,271 solid tumors sequenced with the MSK-IMPACT clinical assay concluded that about 1/3 of patients harbor a standard-of-care predictive biomarker.^2^ Although this estimation is promising, it still leaves the remaining 2/3 of patients despaired by the progress due to the lack of adequate molecular lesions that can be targeted. The landscape becomes even less optimistic considering the multitude of mechanisms by which drug resistance unavoidably emerges following targeted therapies.^3^ ^,^ ^4^ Thus, novel therapeutic approaches must be developed that can be effective in patients exhibiting broader profiles of predictive biomarkers and druggable lesions.

Chemokines and their receptors emerge as promising targets for such therapeutic approaches because they are widely expressed by cancer and stromal cells; however, the overlapping activities, expression profiles, and receptor affinity limit their application for cancer management.^5^ ^,^ ^6^ We have hypothesized that the high redundancy and overlapping profiles between ligands and receptors, instead of a limitation, may be converted to an advantage that can be leveraged therapeutically in developing therapeutic strategies that are effective, irrespective of the specific molecular signature of individual cancers. This could be attained by targeting their cellular targets instead of inhibiting specific activities. To that end, we have linked diphtheria toxin (DT) to the chemokines CCL2 and CCL8. DT is a broad-spectrum cytotoxic agent that is effective against different tumors and is increasingly used in experimental cancer therapeutics.^7-9^ Conjugates of DT and the cytokines IL-2 or IL-3 have been successfully used for the treatment of hematological malignancies, while a specific IL-3 conjugate has been approved for cancer management, and the corresponding drug was designated *Tagraxofusp.* ^10-13^ CCL8 is a chemokine that is overexpressed in both the cancer cells and the cells of the tumor stroma and is thus anticipated to effectively guide DT into the tumors.^14-18^ Indeed, a CCL8-based DT analog, designated DTCCL8, was effective against primary mouse breast cancers triggered by the middle T antigen of the polyoma virus and significantly suppressed their growth in mice.^19^ Cytotoxic analogs based on CCL2 have not been developed so far.

The availability of cytotoxic analogs bearing different homing chemokines may provide means to target a broader spectrum of cancers. Moreover, since different tumors exhibit distinct expression profiles of chemokines and their receptors, it is plausible that the affinity of chimeric peptides against different cancers, and therefore their anticancer activity, could be predicted by assessment of their uptake by cancer cells ex vivo, in cultured explants of primary cancers. We tested these hypotheses by developing DTCCL2, which is structurally related to DTCCL8 but instead of CCL8, bears CCL2 as a homing peptide. CCL8 functions by binding to receptors that include CCR2, CCR3, CCR5, and CCR8, while CCL2 primarily interacts with CCR2.^20^ ^,^ ^21^ The expression of these receptors is upregulated in cancer cells and cells of the tumor stroma, pointing to the targeted delivery of the cytotoxic peptide into the tumor. We compared the uptake of both DTCCL8 and DTCCL2 in cultured tumor explants ex vivo before and after therapy with the cytotoxic analogs and evaluated their anticancer activity in vivo in different human breast cancer models. Our results suggest that cytotoxic chemokine analogs similar to DTCCL2 and DTCCL8 can be used for the management of breast cancer and other cancers. Moreover, they point to the development of an oncology platform by which specific cytotoxic peptides can be matched to individual cancers based on their specific uptake by the tumors for the selection of the most promising for cancer management.

## Results

### Development of DTCCL2

**Figure 1.**
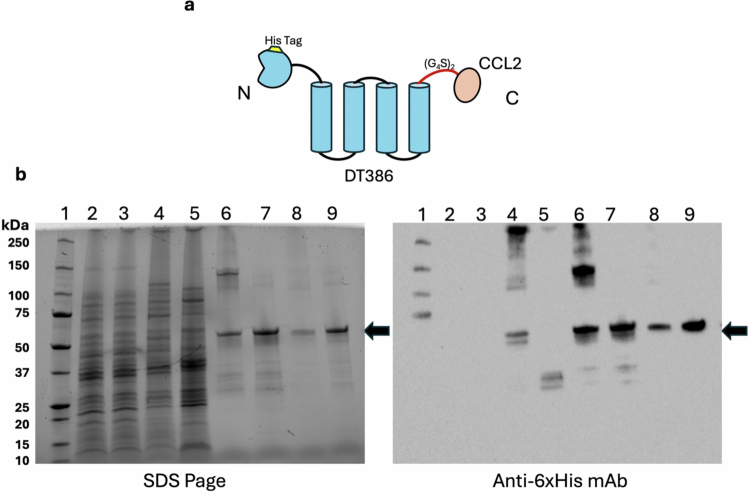
Cytotoxic CCL2 development. (a) The fusion protein is composed of the catalytic and translocation domains of diphtheria toxin (DT386) fused to the human chemokine CCL2 via a flexible linker. The position of an N-terminal His tag is indicated. (b). SDS‒PAGE (left) and immunoblot using an anti-6xHis monoclonal antibody (right). Lanes are defined as follows: (1) molecular weight ladder, (2) uninduced *E. coli* BL21 (DE3) lysate, (3) IPTG-induced lysate, (4) soluble protein, (5) purified DTCCL8 (size control) and (6–9) purified DTCCL2 elution fractions. The arrow shows a prominent band with the expected molecular weight of ~52.24 kDa for DTCCL2.

DTCCL2 was produced by recombinant DNA technology and purified as described in the Methods. The peptide structure is shown in Figure 1a. A His-tag was introduced at the N-terminal while diphtheria toxin (DT, DT386) and the CCL2 homing peptide sequence were connected by a poly-glycine-serine (G4S) link. SDS‒PAGE of protein extracts of unpurified and purified are shown in the left panel of Figure 1b, while anti-His immunoreactivity is shown in the right panel of Figure 1b. DTCCL2 is indicated by an arrow.

### Cytotoxicity of DTCCL2 in vitro

**Figure 2.**
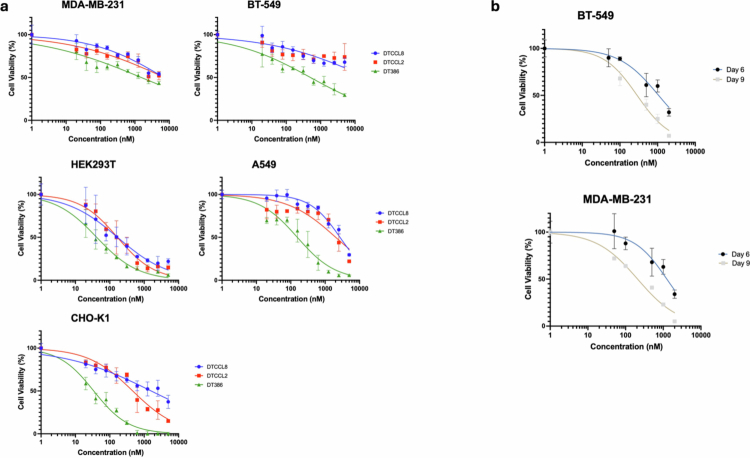
Cytotoxic activity of DTCCL2 and DTCCL8 in vitro. (a). Survival of human TNBC (MDA-MB-231, BT-549), non-small cell lung cancer (A549), and noncancer cell lines (HEK293T and CHO-K1) following 72 h of exposure to varying concentrations (0–5000 nM) of DTCCL2 (red), DTCCL8 (blue) and DT386 (green). The data represent the percentage of live cells relative to the untreated controls and are expressed as the mean ± SD of triplicate wells (*n* = 3). (b). Survival of BT-549 and MDA-MB-231 cells following treatment with DTCCL2 at concentrations ranging from 0 to 2000 nM. Cell viability was assessed at days 3, 6 and 9 post treatment. Data are expressed as the mean ± SD of triplicate wells (*n* = 3).

Initially, we tested the cytotoxic activity of DTCCL2 in a panel of cells, including human breast cancer MDA-MB-231 and BT-549, human non-small cell lung cancer A549, human embryonic kidney (HEK) 293 T cells, and ovarian hamster CHO-K1 cells. For comparison, treatment with DTCCL8 was also included. Unconjugated CCL2 and CCL8 did not affect cell viability at concentrations up to 225 nM in BT-549, MDA-MB-231, or HEK293T cells, even though HEK293T cells were sensitive to both DTCCL2 and DTCCL8 at comparable concentrations (IC50 166 and 167 nM, respectively) and to DT386 at 41 nM, confirming that their cytotoxic activity is attributable to the diphtheria toxin component rather than the chemokine moiety (Supplementary Figure S2). As shown in Figure 2a and summarized in Table 1, following 3 d of exposure, DTCCL2 and DTCCL8 were less toxic than unconjugated DT386 across all the cell lines tested, an observation that is consistent with our previous findings for DTCCL8 and likely reflects differences in the mechanism of cellular entry between the chimeric peptide and unconjugated DT.^19^ In MDA-MB-231 and BT-549 cells, neither DTCCL2 nor DTCCL8 achieved 50% inhibition within the tested concentration range at 3 d. In HEK293T cells, DTCCL2 and DTCCL8 exhibited comparable cytotoxicities, with IC50 values of 166 and 167 nM, respectively, which were approximately fourfold higher than those of DT386 (41 nM), which is consistent with the previously reported sensitivities of HEK293T and MDA-MB-231 cells to truncated diphtheria toxin constructs.^22^ In CHO-K1 cells, DTCCL2 showed greater potency than DTCCL8, with IC50 values of 464 and 1593 nM, respectively. Extended treatment of MDA-MB-231 and BT-549 cells with DTCCL2 progressively increased their cytotoxic activity, with IC50 values decreasing from above 5000 nM at 3 d to 1244 nM and 1063 nM at 6 d for MDA-MB-231 and BT-549, respectively, and 208 nM and 286 nM at 9 d (Figure 2B, Table 1). For comparison, 6d of treatment with DTCCL8 and DT386 in BT-549 and MDA-MB-231 cells resulted in IC50 values of 702 and 449 nM for DTCCL8, respectively, with DT386 maintaining greater potency at 114 and 78 nM (Supplementary Figure S3).

**Table 1.** IC50 values of DTCCL2, DTCCL8, and DT386 in MDA-MB-231, BT-549, A549, HEK293T, and CHO cells after 3 d of treatment, and IC50 values of DTCCL2 in MDA-MB-231 and BT-549 cells after 6 and 9 d of treatment.

Cell Line	DTCCL2 IC50​ (3 d), nM	DTCCL8 IC50​ (3 d), nM	DT386 IC50​ (3 d), nM	DTCCL2 IC50​ (6 d), nM	DTCCL2 IC50​ (9 d), nM
**MDA-MB-231**	>5000*	>5000*	1529	1244	208
**BT-549**	>5000*	>5000*	507	1063	286
**A549**	3500	3400	4800	—	—
**HEK293T**	166	167	41	—	—
**CHO**	464	1,593	34	—	—

— Not evaluated.

^*^ IC50 values greater than 5000 nM indicate that 50% inhibition was not achieved within the tested concentration range.

### Uptake of DTCCL2 and DTCCL8 by breast tumor cells

**Figure 3.**
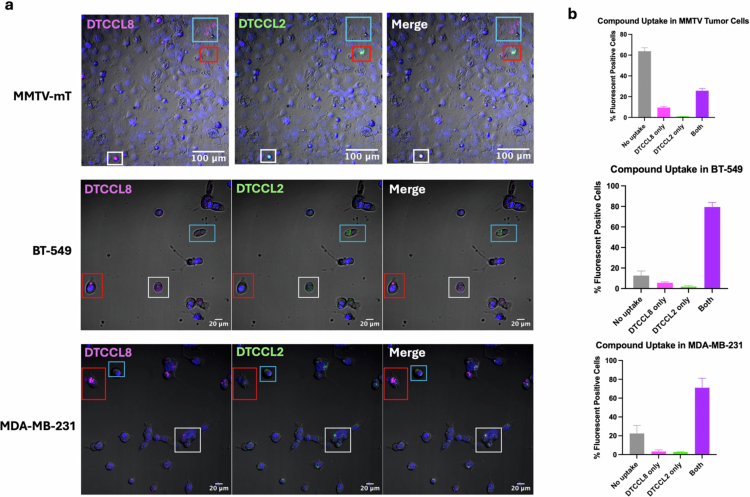
Uptake of DTCCL2 and DTCCL8 by breast tumor cells. (a) Microphotographs of untreated primary tumor cells isolated from MMTV-mT mouse tumors and established human TNBC cell lines (BT-549 and MDA-MB-231). Cells were simultaneously treated with 100 nM of Dylight 488-labeled DTCCL2 (green) and Dylight 633-labeled DTCCL8 (magenta). Nuclei were counterstained with DAPI (blue). The three panels represent the same field of view displayed as separate fluorescence channels: DTCCL8 channel only (left), DTCCL2 channel only (middle) and merged overlay of both channels (right). Colored boxes highlight representative individual cells demonstrating distinct uptake patterns: red boxes indicate cells positive for DTCCL8 only, cyan boxes indicate cells positive for DTCCL2 only, and white boxes indicate cells positive for both peptides. Scale bars represent 100 μm (MMTV-mT) and 20 μm (BT-549 and MDA-MB-231). (b) Quantitative assessment of toxin uptake. Bar graphs represent the percentage of fluorescence-positive cells within the total population. Quantification was performed by analyzing triple-panel fields of view across duplicate wells for each condition. Data are expressed as the mean percentage ± SD (*n* = 2 independent wells).

Subsequently, we tested the uptake of DTCCL2 and DTCCL8 by breast cancer cells. To that end, explants of MMTV-middle T (mT) primary mouse breast tumors and MDA-MB-231 and BT-549 breast cancer cells were exposed to DTCCL2 or DTCCL8 have been labeled with fluorescent dyes. MDA-MB-231 and BT-549 tumor explants exhibited high positivity for both DTCCL2 and DTCCL8, about 70%–80%, while about 13% of the BT-549 and 23% of the MDA-MB-231 cells did not uptake any of DTCCL2 or DTCCL8 (Figure 3). The cells that exhibited positivity for DTCCL2 or DTCCL8 alone were less than about 6% (Figure 3). Thus, in vitro, these breast cancer cells exhibit heterogeneous ability to uptake the cytotoxic peptides with their ability to do so, being redundant, implying overlapping expression profiles for the corresponding receptors.

In MMTV-mT tumor explants, more heterogeneous profiles were recorded, with about 64% of the cells uptaking none and 26% of the cells uptaking both DTCCL2 and DTCCL8 (Figure 3). Moreover, single cytotoxic peptide positivity was as high as 10% for DTCCL8 alone but as low as 1% for DTCCL2 (Figure 3). It is plausible that this is due to a combination of reasons, including the highly diverse cancer cell expression profiles of primary breast tumors and the presence of stromal cells that express differentially specific receptors or no CCL8 or CCL2 receptors.

### DTCCL2 and DTCCL8 inhibit the growth of human breast tumor in mice

**Figure 4.**
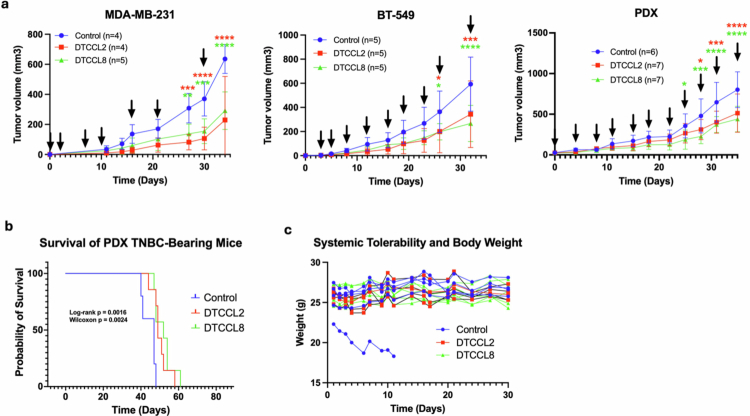
*In vivo* antitumor efficacy of DTCCL2 and DTCCL8 in breast tumor-bearing mice. (a) Therapeutic efficacy of DTCCL2 (red) and DTCCL8 (green) was evaluated in female mice bearing MDA-MB-231 (left, PBS *n* = 4, DTCCL2 *n* = 5, DTCCL8 *n* = 5) or BT-549 (middle, *n* = 5 per group) cell line-derived xenografts and human triple-negative breast PDX (right, *n* = 6–7 per group). The cytotoxic analogs were administered as indicated by the arrows. Data are presented as the mean ± SD. Statistical significance was determined via two-way ANOVA followed by Tukey's multiple comparison test (*p < 0.05; **p < 0.01; ***p < 0.001, and ****p < 0.0001). The color of the asterisks reflects the significance of the corresponding cytotoxic analog (red, DTCCL2; green, DTCCL8). (b) Kaplan‒Meier survival curves for mice bearing human TNBC PDX tumors following treatment with DTCCL2 or DTCCL8. Median survival time was 46 d for the control group, 49 d for DTCCL2, and 52 d for DTCCL8. Median survival was compared between the treatment groups and the PBS control using Log–rank test (*p* = 0.0016) and the Wilcoxon test (*p* = 0.0024). (c) Body weight measurements of individual mice from the MDA-MB-231 cohort treated with DTCCL2, DTCCL8, or the PBS control.

The high uptake of DTCCL8 and DTCCL2 by human cancer cells and tumor explants in vitro, combined with the earlier reported effective treatment of mouse breast cancers by DTCCL8,^19^ prompted us to explore the anticancer activity of the cytotoxic peptides in human breast cancers in vivo. Therefore, nude mice bearing triple-negative breast cancer (TNBC) BT-549 or MDA-MB-231 human breast cancer cells and NSG mice bearing PDX TM00096 from primary human breast cancer (TNBC ER-/PR-/HER2-) were treated with cytotoxic peptides every 3‒5 d, and the tumor volume was evaluated after about 5 weeks. Both drugs produced significant anticancer activity, inhibiting tumor growth by more than 50% after about 5 weeks of treatment. The growth of MDA-MB-231 xenografts was inhibited by DTCCL8 by 65% and by 55% by DTCCL2. BT-549 xenografts were more sensitive to DTCCL8 (~55% tumor growth inhibition), followed by DTCCL2 (~40% inhibition). The human breast tumor PDX was inhibited by DTCCL2 by about 35% and by DTCCL8 by 55% (Figure 4a). Significant tumor growth inhibition (*p* < 0.05 or greater, two-way ANOVA) was attained by both cytotoxic peptides in all three models after 3–4 weeks (Figure 4a), but in neither model was the difference significant between the two treatment groups. Moreover, in the PDX model, the tumors were allowed to grow beyond 35 d and were sacrificed when the animals became moribund or when the tumors exceeded large to construct a pseudosurvival curve, which indicated that treatment significantly attenuated the animals' survival (Figure 4b). DTCCL8 treatment appeared to be moderately more effective than DTCCL2 treatment, but the difference was insignificant.

Both drugs were well tolerated by the tumor-bearing mice, as indicated by the lack of noticeable loss of body weight (Figure 4c).

### Cytotoxic peptide uptake prior and after treatment

**Figure 5.**
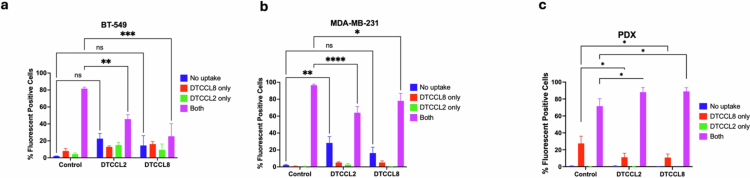
Quantification of targeted toxin internalization in primary tumor explants following in vivo treatment. Control groups represent explants from untreated tumor-bearing mice. (a–c) Differential uptake of DTCCL2 and DTCCL8 in cultured explants from the MDA-MB-231 (a), BT-549 (b), and PDX (c) models. Following 24 h of co-treatment with 100 nM fluorescently labeled toxins, automated single-cell analysis was performed using the StarDist deep learning pipeline. Stacked bars represent the percentage of the total cell population positive for DTCCL2 only, DTCCL8 only, both toxins, or no uptake. Data are expressed as the mean ± SD of independent tumors per treatment group. Tumor explants from three independent tumors per treatment group were analyzed. Measurements were obtained from duplicate wells per tumor, with each well representing the average of three representative fields.

Subsequently, we tested the uptake of DTCCL2 and DTCCL8 in cultured explants of the MDA-MB-231 and BT-549 tumors and in the human breast cancer PDX (Figure 5). In control tumors (untreated), both the MDA-MB-231 and BT-549 explants showed high, above 80% positivity for both DTCCL8 and DTCCL2, which is broadly consistent with the high uptake observed in the same cell lines cultured in vitro (Figure 3), though a direct quantitative comparison between in vitro and ex vivo conditions was not performed. Positivity for neither or either cytotoxic peptide alone was minimal (Figure 5). The PDX also exhibited high positivity for both peptides, above 70%, and exhibited uptake for DTCCL8 alone close to 30%, while for DTCCL2 alone and neither cytotoxic peptide, the positivity was minimal.

Following treatment, the results were not consistent between the different tumors and the cytotoxic peptides, probably because of the diversity of the cells that express the corresponding receptors. In MDA-MB-231 and BT-549 tumors, treatment with either DTCCL2 or DTCCL8 significantly reduced the fraction of cells that were positive for both cytotoxic peptides, implying depletion of the cells that expressed the DTCCL2 and DTCCL8 receptors. This effect was more prominent for BT-549. Of note, however, is that in the PDX model, the treatment moderately but significantly increased the cells that could uptake the cytotoxic peptides.

Positivity to either DTCCL8 or DTCCL2 alone increased moderately after the treatments in MDA-MB-231 and especially in BT-549 tumors. In the PDX model, however, treatment with either DTCCL2 or DTCCL8 significantly reduced the number of cells that could uptake DTCCL8 only, while exclusive DTCCL2 positivity remained undetectable in the tumors of either the control or the treated animals.

### Nuclei shrinkage following treatment

**Figure 6.**
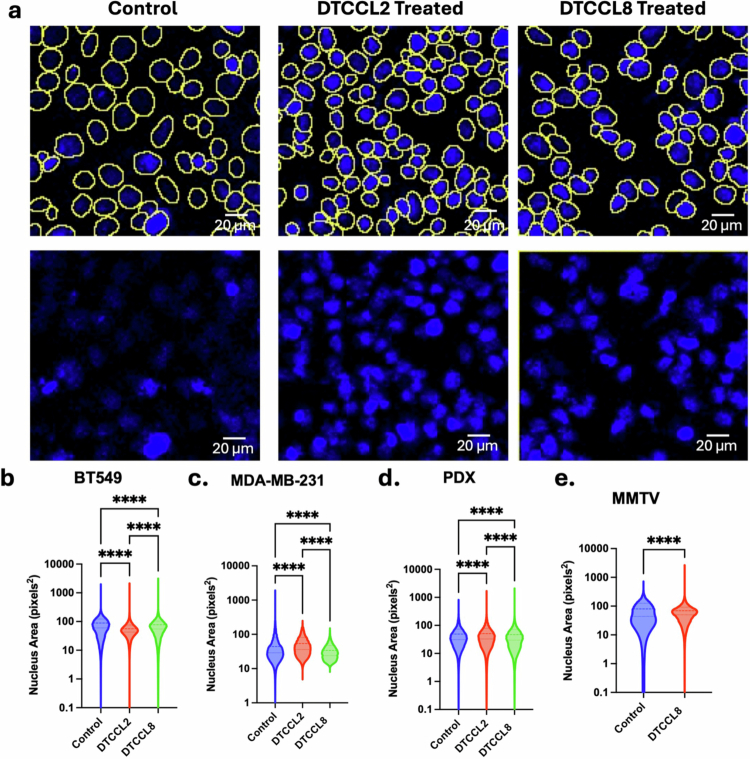
*In vivo* treatment with diphtheria toxin conjugates reduces the nucleus area in tumor explants. (a) Representative confocal microscopy images of DAPI-stained nuclei from BT-549 tumor explants. Explants were derived from mice previously treated with PBS (control), DTCCL2, or DTCCL8. Yellow outlines represent automated nuclear segmentation generated via the StarDist model within the Napari interface Scale bar = 20 µm. (b–e) Violin plots showing the distribution of the nucleus area (pixel²) in explant cultures from four different breast cancer models: BT-549 (b), MDA-MB-231 (c), PDX (d), and MMTV-PyMT (e). Explants were derived from mice treated *in vivo* with 1.5 mg/kg of the indicated compounds. For the BT-549, MDA-MB-231, and MMTV models, analyses were performed using explants from three independent tumors per treatment group, whereas the PDX model included five independent tumors per group. Violin plots represent pooled measurements from thousands of individual nuclei obtained from representative imaging fields. Statistical comparisons were performed using the Kruskal‒Wallis test followed by Dunn's multiple comparisons test (b–d) or the Mann‒Whitney *U* test (e). Asterisks (****) indicate *p* < 0.0001 compared to the corresponding control group.

Nuclei shrinkage or pyknosis represents a common manifestation of apoptotic cell death.^2-25^ We tested this phenomenon by analyzing the nuclear size of the cultured tumor explants from this and previous studies.^19^ As noted in Figure 6, we noted a small but significant reduction in average nuclear size in all tumors that received either DTCCL8 or DTCCL2 compared to the controls.

### Annexin V/PI flow cytometry confirms apoptotic cell death

To further confirm that the reduction in cell viability observed via XTT assay reflects cytotoxicity rather than cytostatic activity, Annexin V/PI flow cytometry was performed in MDA-MB-231 and BT-549 cells treated with DTCCL2 or DTCCL8 at days 3, 6, and 9. Both compounds induced progressive increases in early apoptotic (Annexin V + /PI-) and late apoptotic (Annexin V+/PI+) populations over time in both cell lines, while untreated control cells maintained high viability throughout the observation period. These findings confirm that DTCCL2 and DTCCL8 induce apoptotic cell death and validate the observations of nuclear shrinkage reported above. Representative dot plots and quantification data are shown in Supplementary Figure S4.

### Effects of treatment in myeloid cells in vivo

**Figure 7.**
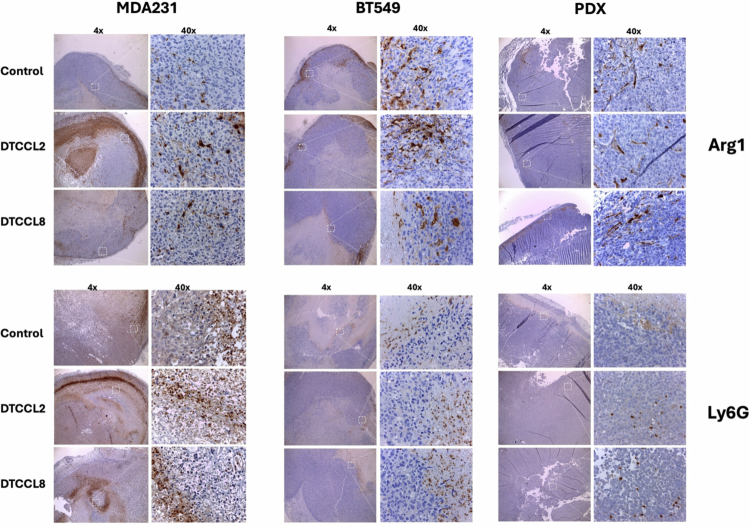
Myeloid cell expression in tumors. Immunohistochemical (IHC) staining for the markers Arg1 and Ly6G in MD-MB-231, BT-549, and PDX tumor sections. Tumors were harvested from mice following treatment with PBS control or DTCCL2 or DTCCL8. Positive staining was indicated by a brown DAB chromogen signal against blue hematoxylin nuclear counterstain. For each condition, the right panels indicate enlarged (40×) images of the region of interest (white dashed box) shown in the corresponding (4×) field on the left.

Given the expression of the receptors for CCL2 and CCL8 in stroma cells, especially those of myeloid linages,^26^ ^,^ ^27^ we evaluated the expression of arginase 1 (Arg1) and Ly6G in tumors without or with cytotoxic peptide treatment. Arg1 is a widely used marker for the pro-oncogenic/anti-inflammatory M2 differentiation of macrophages, while Ly6G is a marker for neutrophils.^28-32^ As shown in Figure 7, positivity for both Arg1 and Ly6G in MDA-MB-231 cells and Arg1 in BT-549 cells was mainly limited to the tumor front. In the PDX model, positivity was minimal (for Arg1) or absent (for Ly6G), likely due to the severe immunodeficiency of the NSG mice. Treatment did not affect Arg1 or Ly6G immunopositivity.

## Discussion

In an effort to provide a strategy for tumor targeting by a manner that is not restricted by the specific molecular lesions that are present in the tumors, we leveraged the abundant expression of receptors in the tumors that are recognized by CCL8 and CCL2 chemokines. These chemokines are expressed by both cancer and stromal cells and mediate their activities via interactions with receptors that primarily include CCR2 and CCR5 in the breast cancer context, among others.^14^ ^,^ ^33-37^ To that end, we have developed cytotoxic conjugates that consist of these chemokines and diphtheria toxin, a highly toxic peptide exhibiting broad specificity,^7-9^ and tested them against a panel of human breast cancer lines growing in mice and a PDX model from TNBC. Our results showed significant anticancer activity against all three tumor models and increase in the animals’ survival in the PDX model in vivo. Receptor-mediated cytotoxicity of DTCCL8 was previously demonstrated through CCR5 overexpression and antibody blocking experiments, where transient overexpression of CCR5 resulted in greater cell death with DTCCL8 compared to DT386, and pretreatment with an anti-CCR5 blocking antibody reduced DTCCL8 cytotoxicity without affecting DT386 activity.^19^ Furthermore, DT386 administered at 1.5 mg/kg was acutely toxic in mice while DTCCL8 at the same dose was well tolerated, demonstrating that the chemokine-targeting domain is essential for in vivo selectivity.^19^

Notably, the comparison in cytotoxic peptide uptake by MDA-MB-231 and BT-549 cells growing in vitro vs the same cells growing in vivo as xenografts in nude mice. Positivity appeared higher in tumor explants from mice compared to cells cultured in vitro, suggesting potential upregulation of chemokine receptors, though a formal direct quantitative comparison was not performed (Figure 3b and Figure 5). The recorded increase in positivity is due to the presence of stromal cells that are absent in vitro, which is unlikely because the amount of staining for Arg1 and Ly6G in the tumors, which are expressed by macrophages and neutrophils that express the corresponding receptors, was minimal (Figure 7). This observation underscores the divergence of the expression signatures of cancer cell lines in vitro from those in vivo when the expression of chemokine receptors is evaluated. This finding is also consistent with the sensitization of the signaling networks that mediate cell-to-cell communication in vivo, such as those involving chemokines and their receptors, as opposed to the conditions of in vitro growth in cell culture.

The high uptake of the cytotoxic analogs in vivo in tumor explants is in line with their strong antitumor activity, which significantly inhibited tumor growth in mice and prolonged their survival in the PDX model. This finding is also consistent with the moderate but significant nuclei shrinkage induced by the treatment in all the models, which is indicative of sensitization to the induction of cell death. Evaluation of the uptake of the cytotoxic peptides in the tumors that did not receive treatment and a comparison with the treated tumors showed diverged results between the cancer cell lines and the PDX model. In the two cancer cell xenografts, the treatment significantly reduced the number of cells that could uptake both peptides but moderately increased the number of cells that uptook each of the cytotoxic peptides alone, which is likely due to clonal selection following treatment. The opposite trend, however, was unveiled in the PDX model, in which, following treatment, the fraction of cells that were positive for both peptides increased, accompanied by a decrease in the number of cells uptaking DTCCL8 only. It is plausible that the treatment has converted DTCCL8-uptaking positive cells to DTCCL8 and DTCCL2 positive cells, hence increasing the total fraction of cells with dual uptaking in expense of the cells uptaking only DTCCL8. This observation was evident only in the PDX model. It is plausible that the PDX model, which resembles more accurately the naturally occurring tumors, is characterized by high plasticity and cellular heterogeneity, which enables the selection of different clonal populations. In the BT-549 and MDA-MB-231 models, which exhibit higher positivity for both peptides, clonal selection was more limited.

While the activity of the cytotoxic peptides through interaction with stromal cells cannot be ruled out, it is rather unlikely to present the main mechanism of their activity. The minimal presence or lack of neutrophils and M2 macrophages by all tumors that, when present, were limited to the tumor margins argues in favor of the direct activity of the cytotoxic peptides on the cancer cells, without, however, precluding activity in stromal cells when present, other tumor models.

Several limitations of this study should be acknowledged. The in vitro evaluation was limited to two TNBC cell lines, and future studies should expand to a broader panel including nontransformed cells to more fully characterize the therapeutic window of these constructs. Potential immunogenicity and pre-existing anti-diphtheria toxin immunity due to prior vaccination represent translational considerations for DT-based therapeutics. However, clinical experience with denileukin diftitox, an FDA-approved DT fusion protein, demonstrated that pre-existing anti-DT antibodies present in the majority of treated patients did not reliably predict a lack of clinical response,^38^ suggesting that vaccination may not fully disrupt therapeutic efficacy. Binding of DTCCL2 and DTCCL8 to CCR2- and CCR5-expressing normal blood immune cells represents an additional off-target consideration that warrants further investigation.

Several approaches could potentially improve the efficacy of DTCCL2 and DTCCL8 in these and other models. Combination with standard chemotherapeutic agents would present a promising strategy, as the CCL5/CCR5 axis has been shown to mediate cisplatin resistance through CAF-derived CCL5-mediated activation of AKT signaling in tumor cells, and pharmacologic inhibition of this axis sensitized tumor cells to cisplatin and prolonged survival in preclinical models.^39^ This suggests that DTCCL8, which targets CCR5, could synergize with platinum-based chemotherapy by eliminating CCR5-expressing tumor cells and disrupting resistance signaling. In the PDX model specifically, the observed increase in dual receptor positivity following treatment suggests that simultaneous or sequential targeting of both CCR2 and CCR5 could further improve outcomes by limiting escape through receptor plasticity. Finally, an intriguing possibility would be to pretreat with CCL2/8 prior to the administration of cytotoxic peptides to upregulate the corresponding receptors and improve their efficacy.

Collectively, the present study demonstrated that the use of cytotoxic peptide analogs based on CCL-like chemokines represents a promising strategy for the management of breast and other cancers. Owing to the high heterogeneity of primary tumors in terms of the profile of expression of receptors for different chemokines, it is plausible that the availability of different cytotoxic peptide conjugates may be used to develop personalized therapies. A proof of principle of this strategy has been provided here, demonstrating the feasibility of testing tumors ex vivo for cytotoxic analog uptake and then selecting the most adequate moiety for treatment.

## Materials and methods

### Design and construction of DTCCL2 and DTCCL8 fusion toxins

The design and construction of the chimeric fusion toxins were performed as previously described.^19^ Briefly, the DT386-hCCL8-6xHis and DT386-hCCL2-6xHis constructs were synthesized by GenScript and cloned into the pET30a(+) backbone. Both constructs share a similar structure, where the catalytic and translocation domains of diphtheria toxin (DT386) are connected to the human chemokine ligand (CCL8 or CCL2) via a flexible (G_4_S)_2_ linker. An N-terminal 6xHis tag was included for affinity purification. The truncated control, DT386, was generated using splicing overlap extension PCR (SOEingPCR) as previously detailed.^19^

### Expression, purification, and characterization of fusion toxins

The expression and purification of DTCCL2, DTCCL8, and the truncated DT386 control were performed using our previously established protocols.^19^ Briefly, the plasmids were transformed into BL21 (DE3) *E. coli*, and the cultures were grown at 37 °C until they reached an OD_600_ of 0.7. Plasmids were verified via sequencing. The sequence of DTCCL8 was reported in ref. 19. and the sequence of DTCCL2 is shown in Supplementary Figure S1. Protein expression was induced with 0.3 mM IPTG (Thermo Fisher Scientific, Cat. No. 15529019) for 3 h. Following cell harvesting and lysis, the inclusion bodies containing the chimeric proteins were solubilized and refolded over 72 h at 4 °C in refolding buffer (50 mM Tris/HCl, pH 8.5, 0.4 M sucrose, 10% glycerol, 0.5% Triton X-100, 0.3 mM GSSG, and 3 mM GSH). The refolded proteins were purified via Ni-NTA (Thermo Fisher Scientific, Cat. No. 88221) affinity chromatography and subjected to endotoxin removal using Pierce high-capacity endotoxin removal spin columns (Thermo Fisher Scientific, Cat. No. 88274). For Western blot analysis, samples were separated via SDS‒PAGE on a 12% polyacrylamide gel and transferred to a PVDF membrane. Membrane was blocked with 5% nonfat dry milk in PBST for 1 h at room temperature and then incubated with a mouse 6xHis Tag mAb HRP-conjugated primary antibody (MA1-21315-HRP, Thermo Fisher Scientific) at a dilution of 1:500 for 1 h at room temperature. Signal was detected using enhanced chemiluminescence (ECL). Final protein concentrations were determined via a BCA assay (Thermo Fisher Scientific, Cat. No. A55861).

### Cell culture and maintenance

The human triple-negative breast cancer (TNBC) cell lines MDA-MB-231 (RRID: CVCL_0062) and BT-549 (RRID: CVCL_1092), the lung carcinoma line A549 (RRID: CVCL_0023), and the HEK293T (RRID: CVCL_0045) and CHO-K1 (RRID: CVCL_0214) lines were originally obtained from the American Type Culture Collection (ATCC). Cells were cultured in Dulbecco's modified Eagle's medium (DMEM; Gibco) supplemented with 10% fetal bovine serum (FBS; Gibco) and 1% penicillin-streptomycin (Gibco). CHO-K1 cells were maintained in Ham's F-12 Nutrient Mixture (Gibco) with identical supplements. All the cultures were incubated at 37 °C in a humidified atmosphere containing 5% CO_2_. Human cell lines were validated by short tandem repeat (STR) profiling within the last 3 y via the Functional Genomics Core of the Center for Targeted Therapeutics. All experiments were conducted using mycoplasma-free cells, as confirmed by the MycoAlert Mycoplasma Detection Kit (Lonza, LT07-318).

### *In vitro* cytotoxicity assays

To evaluate the cytotoxic efficacy of DTCCL8, DTCCL2, and the DT386 control, experiments were done across a panel of cell lines, including MDA-MB-231, BT-549, HEK293T, A549, and CHO-K1. Cells were seeded in 96-well plates at a density of 7,000 cells per well and allowed to adhere overnight. Cells were treated with a series of ten concentrations of each toxin variant using serial dilutions ranging from 5000 nM to 9.7 nM. For standard viability assessment, treatments were applied for 72 h. To evaluate the kinetics of cytotoxicity in TNBC lines (MDA-MB-231 and BT-549), cell viability was measured at 3, 6, and 9 d post treatment with concentrations up to 2000 nM. Cell viability was quantified using the CyQUANT XTT Cell Viability Assays (Thermo Fisher Scientific, Cat. No. X12223) according to the manufacturer's instructions. Following a 2 h incubation with XTT reagent at 37 °C, the absorbance was measured at 450 nm (reference wavelength 650 nm) using a microplate reader. All conditions were assessed in triplicate wells, and the results were normalized to those of the untreated control wells.

### Annexin V/PI apoptosis assay

To evaluate apoptotic cell death, MDA-MB-231 and BT-549 cells were seeded at 150,000 cells per well in 6-well plates and treated with 500 nM DTCCL2 or DTCCL8. At days 3, 6, and 9 post-treatment, both floating and adherent cells were collected. Briefly, conditioned media containing floating cells were collected and reserved. Adherent cells were detached using TrypLE Express Enzyme 1X (Thermo Fisher Scientific, Cat. No. 12605010) for 2 min at 37 °C. Both fractions were combined, washed, and stained using the Dead Cell Apoptosis Kit with Annexin V Alexa Fluor 488 and PI (Thermo Fisher Scientific, Cat. No. V13241) according to the manufacturer's instructions. Samples were acquired on a BD LSR II flow cytometer with the HTS option, and the data were analyzed using FlowJo software. Early apoptotic cells were defined as Annexin V+/PI− and late apoptotic cells as Annexin V+/PI+. Singlets were gated prior to apoptosis analysis.

### Fusion toxin fluorescent labeling

Fluorescent labeling of DTCCL8 and DT386 was performed using DyLight 633 NHS Ester (Thermo Fisher Scientific, Cat. No. 46417) as previously described.^19^ For dual-uptake studies, DTCCL2 was similarly labeled with DyLight 488 NHS Ester (Thermo Fisher Scientific, Cat. No. 46403) following the manufacturer's protocol. Excess dye was removed using Pierce Dye Removal Columns (Thermo Fisher Scientific, Cat. No. 22858).

### Internalization assay and confocal microscopy

MDA-MB-231 and BT-549 cells were seeded in 96-well glass bottom plates (Cellvis, Cat. No. P96-1.5H-N) coated with poly-D-lysine (Thermo Fisher Scientific, Cat. No. A3890401) at a density of 10,000 cells per well. Primary tumor cells were isolated from MMTV-PyMT mammary tumors (RRID:IMSR_JAX:002374). Cells were treated for 24 h with 100 nM of labeled DTCCL8, DTCCL2, or a combination of both. Prior to imaging, the nuclei were counterstained with DAPI (Thermo Fisher Scientific, Cat. No. 62248). Imaging was performed using a Zeiss LSM 700 confocal microscope, and the fluorescence intensity was quantified using ImageJ. For each condition, representative fields were captured using consistent laser settings.

### Establishment of cell line-derived xenograft (CDX) models

All animal procedures were conducted in accordance with the ethical guidelines and approved protocols of the Institutional Animal Care and Use Committee (IACUC) at the University of South Carolina (protocol # 2766-102052-071625). All animals were obtained from The Jackson Laboratory (Bar Harbor, Maine). Female NU/J mice (RRID:IMSR_JAX:002019) and NSG mice (RRID:IMSR_JAX:005557) were maintained on a 12-h light/dark cycle with standard chow and water provided ad libitum under specific pathogen-free conditions. Animals were housed in groups in ventilated cages with standard environmental enrichment. For the establishment of cell line-derived xenografts (CDXs), donor tumors were first generated via the subcutaneous injection of 4 × 10^6^ MDA-MB-231 or BT-549 cells (resuspended in 100 μL of a 1:1 PBS/Matrigel mixture) into the flanks of donor mice. Once the donor tumors reached a median size (approximately 500–800 mm^3^), they were harvested and aseptically cut into uniform fragments, which were then transplanted into recipient mice using a trocar needle while under isoflurane anesthesia. For the patient-derived xenograft (PDX) model, human TNBC TM00096 (JAX-PDX) mice (passage P9) were obtained from Jackson Laboratory, and the tumors were pre-engrafted into the mammary fat pads of NSG mice. Animals were euthanized via cervical dislocation under isoflurane anesthesia as recommended by the American Veterinary Medical Association.

### Randomization and blinding

Mice were randomly assigned to treatment groups once the tumors reached palpable size using a random allocation method to ensure comparable mean tumor volumes at treatment start. For histological and image-based analyses, representative fields were selected using a randomized approach. Data analysis was conducted using predefined statistical criteria. No protocol was preregistered for this study.

### Therapeutic treatment and efficacy monitoring

Mice were monitored daily until the transplanted fragments yielded palpable tumors to ensure successful engraftment and consistent growth prior to the initiation of therapy. Upon reaching this threshold, the animals were randomly assigned to treatment groups to receive intraperitoneal (i.p.) injections of PBS vehicle control, DTCCL2, or DTCCL8 at a dosage of 1.5 mg/kg administered twice weekly.

For the CDX models, the study was terminated collectively once the tumors in the control group reached the maximum allowable volume according to IACUC guidelines, at which point all experimental and control mice were euthanized for tissue collection. In contrast, the PDX study was conducted as a survival analysis; each mouse was maintained until its individual tumor reached the predetermined volume endpoint, allowing for the generation of Kaplan‒Meier survival curves. For all in vivo studies (CDX and PDX), humane endpoints were defined in accordance with the approved IACUC protocol as a tumor burden not exceeding 10% of body weight, corresponding to approximately 1500 mm^3^, or earlier if ulceration or signs of distress, including >15% body weight loss or impaired mobility, were observed. Tumor growth was assessed bi-weekly using digital calipers, with volumes (*V*) calculated using the formula *V = 0.5 × L × W* ^*2*^. Systemic toxicity was monitored through regular health assessments and recording of body weight throughout the study period. At the respective study endpoints, tumors were harvested and divided for histological fixation in 10% neutral buffered formalin, snap freezing on dry ice, or primary culture in RPMI 1640.

### Experimental unit and outcome measures

The experimental unit was an individual mouse. The primary outcome measure was tumor growth inhibition, as assessed by tumor volume measurements. Secondary outcomes included survival, body weight monitoring, histological analysis, and *ex vivo* toxin internalization efficiency.

### Primary tumor dissociation and cell isolation

Freshly harvested tumors from MMTV-PyMT, human CDX, and human PDX models were collected in sterile RPMI 1640 medium supplemented with 1% penicillin‒streptomycin. The tissue was mechanically dissociated into fine pieces using a sterile blade and subsequently digested in 2 mg/mL Collagenase IV (Thermo Fisher Scientific, Cat. No. 17104019) for 45 min at 37 °C. The enzymatic digestion was neutralized by the addition of RPMI 1640 containing 10% fetal bovine serum (FBS). The suspension was passed through a 70 μm cell strainer to obtain a single-cell suspension. Cells were pelleted via centrifugation at 300 × *g* for 5 min, resuspended in fresh RPMI 1640 with 10% FBS, and cultured on poly-D-lysine-coated 96-well glass-bottom plates (Cellvis, Cat. No. P96-1.5H-*N*) for 24–72 h to establish stable morphology prior to experimental use.

### Quantitative image analysis

To ensure objective and high-throughput quantification, automated image analysis was implemented using the Napari interface. For all the confocal datasets, including monolayer cultures and tumor explants, individual nuclei were segmented from the DAPI channel using a pretrained StarDist deep learning model to generate nuclear masks. StarDist, which uses a convolutional neural network to predict star convex polygons, was used for its ability to resolve individual nuclei in crowded environments. To determine toxin internalization and delivery efficiency, the mean fluorescence intensity (MFI) of the DyLight 488 (DTCCL2) and DyLight 633 (DTCCL8) channels was measured within each nuclear region of interest (ROI) via ImageJ. Positivity thresholds for each toxin were manually established by visual comparison of fluorescence intensity in clearly positive versus negative cell populations. For uptake assays in tumor explants, two independent wells were analyzed per tumor, with percentages derived from three representative fields per well. Final results were calculated as the mean percentage of positive cells across these technical replicates and are expressed as the mean ± SD. In parallel, the same nuclear masks were used for high-resolution morphometric analysis. The area and perimeter of every segmented nucleus were measured to characterize structural changes related to toxin-induced cytotoxicity. To capture the full landscape of the tumor response, single-cell measurements from thousands of individual nuclei were pooled across all representative fields to generate population distributions for each treatment group. For the MMTV, BT-549, and MDA-MB-231 models, measurements were derived from three independent tumors per treatment group, with one explant culture well analyzed per tumor and three independent imaging fields acquired per well. For the PDX model, explants from five independent tumors per treatment group were analyzed, with one culture well examined per tumor. In all cases, single-cell measurements from the sampled fields were pooled to generate populations for each treatment group.

### Immunohistochemistry

At the study endpoint, harvested tumors from the CDX and PDX models were fixed in 10% neutral buffered formalin and embedded in paraffin. Tissue sections were deparaffinized in xylenes and rehydrated through a graded series of ethanol. Heat-induced epitope retrieval was performed using citrate buffer (pH 6.0) in a steamer for 20 min. To ensure high sensitivity while eliminating background from endogenous mouse IgG, the sections were processed using the Vector® M.O.M.® ImmPRESS® HRP Polymer Kit (Vector Laboratories, Cat. No. MP-2400) according to the manufacturer's instructions. Following blocking, the sections were incubated for 1 h at room temperature with primary antibodies against Arg1 (ABclonal, Cat. No. A25808) at a dilution of 1:5000 and Ly6G (ABclonal, Cat. No. A22270) at a dilution of 1:5000. Target proteins were visualized using the ImmPRESS-HRP polymer reagent (Vector Laboratories, Cat. No. MP-7801-15) followed by the included DAB (3,3'-diaminobenzidine) chromogen substrate. Sections were counterstained with hematoxylin to visualize the nuclear morphology. Slides were dehydrated, cleared, and mounted for brightfield microscopy.

### Inclusion and exclusion criteria

All animals that developed palpable tumors were included in the study, with the exception of one control mouse in the MDA-MB-231 cohort that was removed from the study due to rapid weight loss unrelated to treatment. No other animals were excluded from the analysis, and no data points were removed from the statistical analysis.

### Statistical analysis

Statistical analyses were performed using GraphPad Prism (v10.0). For all experimental datasets, including cell viability, in vivo tumor growth and ex vivo internalization assays, the results are expressed as the mean ± SD. Comparisons between multiple treatment groups were assessed using a one-way or two-way ANOVA followed by Tukey's post hoc test for multiple comparisons. Survival data were analyzed using the Kaplan–Meier method, and differences between groups were evaluated via the log rank (Mantel–Cox) test. For morphometric distributions of the nuclear area and perimeter, differences were assessed using the Mann‒Whitney *U* test or Kruskal‒Wallis test to account for nonnormal distributions in large single-cell datasets. Data distribution was assessed prior to parametric testing, and nonparametric analysis was applied when normality assumptions were not met. In all analyses, *p* values <0.05 were considered statistically significant. Sample sizes for the in vivo experiments ranged from *n* = 4–7 mice per group, as specified in the figure legends. Sample sizes were determined based on prior in vivo tumor studies conducted in our laboratory, including MMTV tumor implantation models, which demonstrated that group sizes within this range were enough to detect biological differences in tumor growth.

## Supplementary Material

Figure S4.jpg Figure S4.jpg

Figure S2.jpg Figure S2.jpg

Figure S3.jpg Figure S3.jpg

Figure S1.jpg Figure S1.jpg

## Data Availability

The datasets generated during this study are publicly available in Zenodo repository: 10.5281/zenodo.18943178.
