# Supplementary figures and images for: Effective treatment of human breast tumors by chimeric CCL2 and CCL8 diphtheria toxin cytotoxic peptides
Source: Cancer Biol Ther. 2026 Jul 2;27(1):2688479. doi: 10.1080/15384047.2026.2688479 (PMC13336256; doi:10.1080/15384047.2026.2688479)

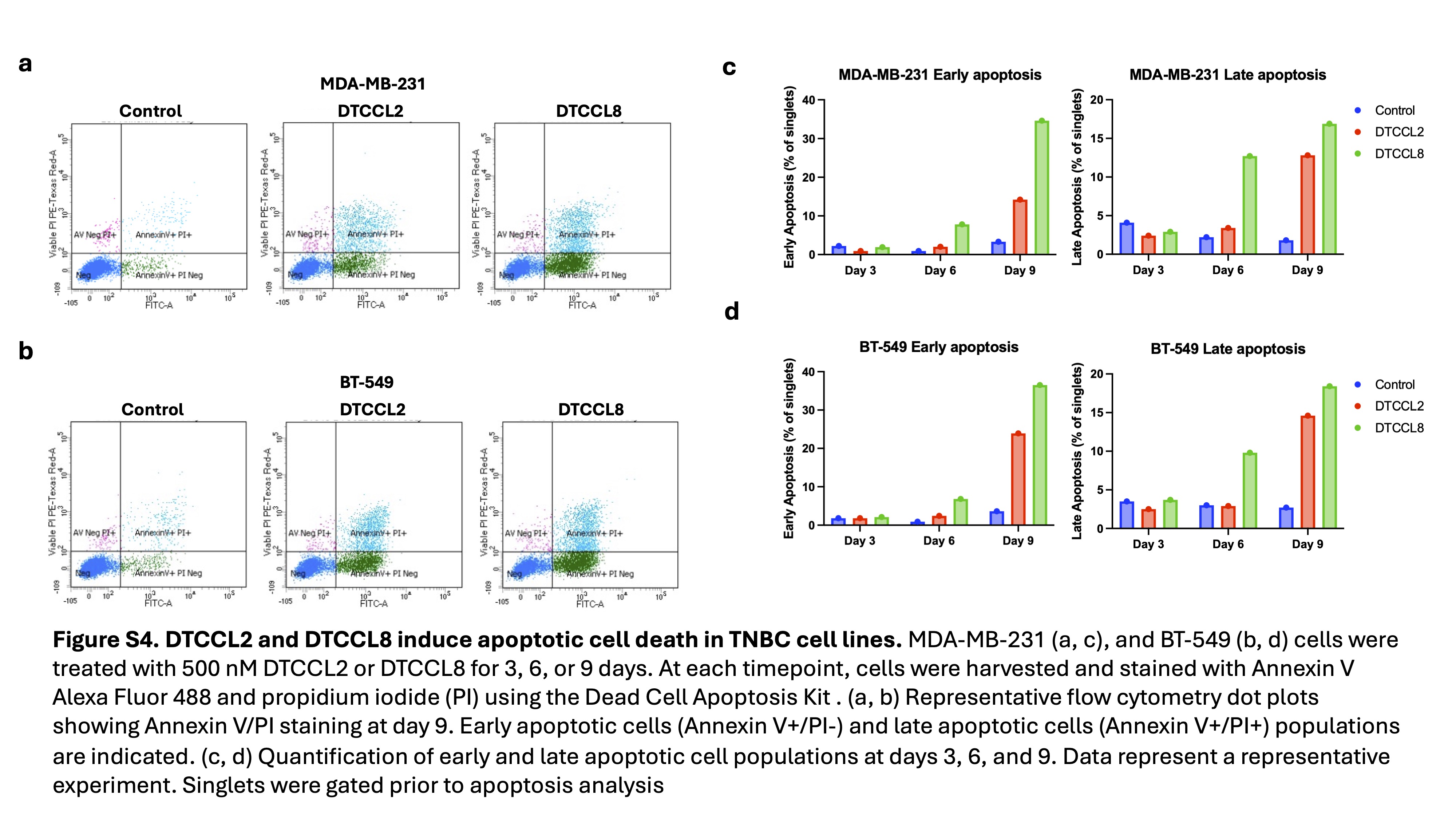

Supplement: Figure S4.jpg [file KCBT_A_2688479_SM7335.jpg]

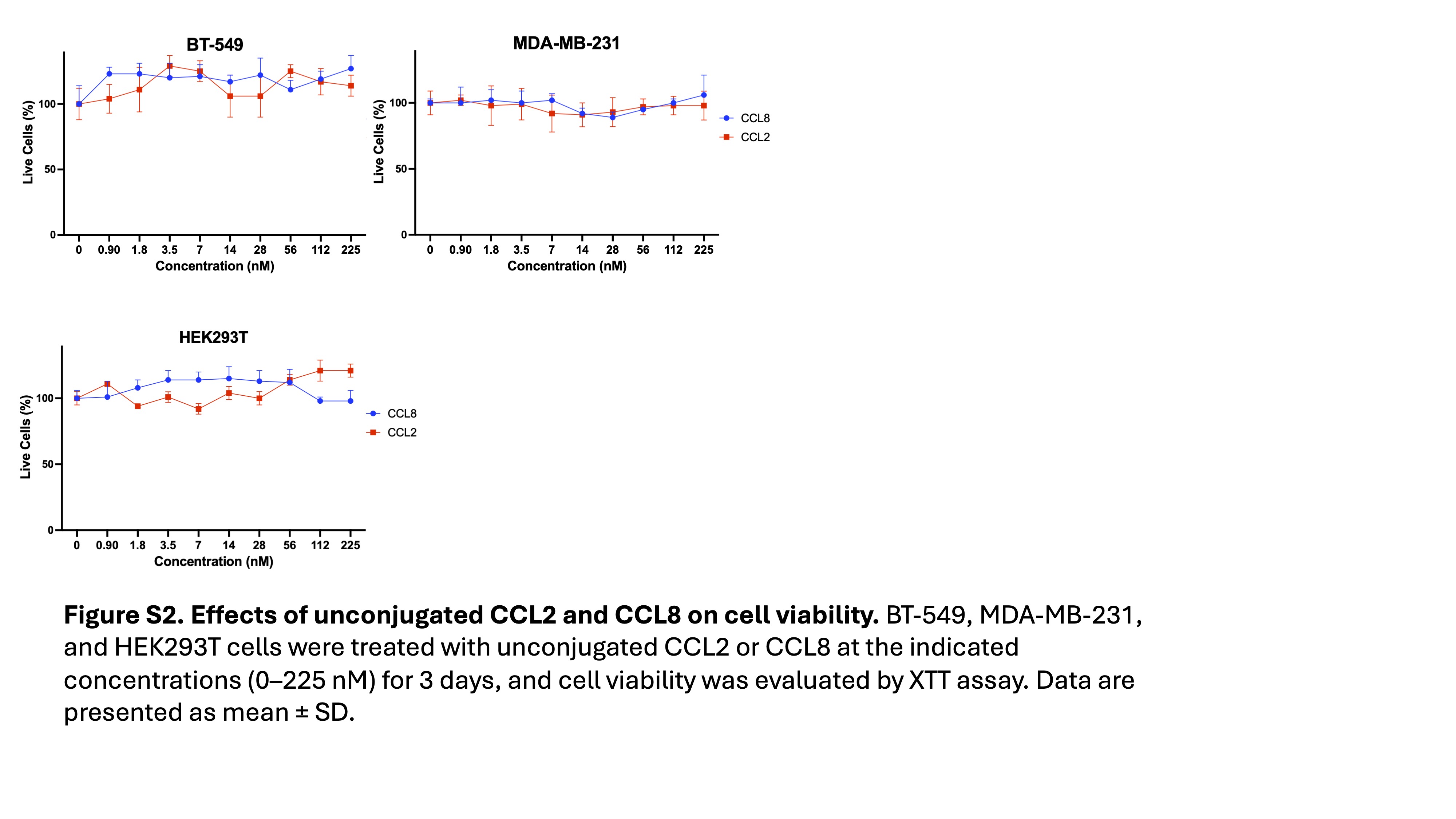

Supplement: Figure S2.jpg [file KCBT_A_2688479_SM7336.jpg]

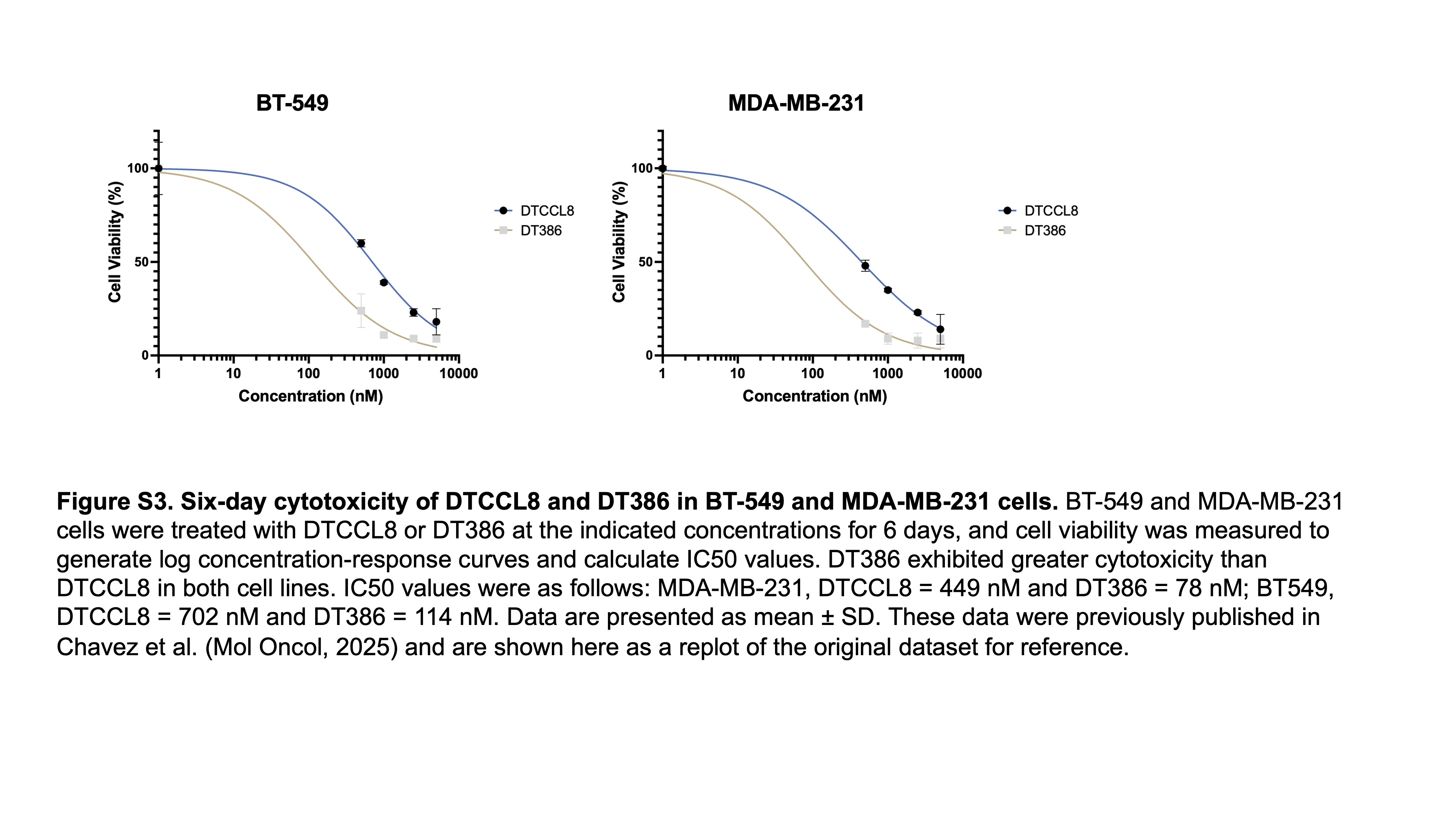

Supplement: Figure S3.jpg [file KCBT_A_2688479_SM7337.jpg]

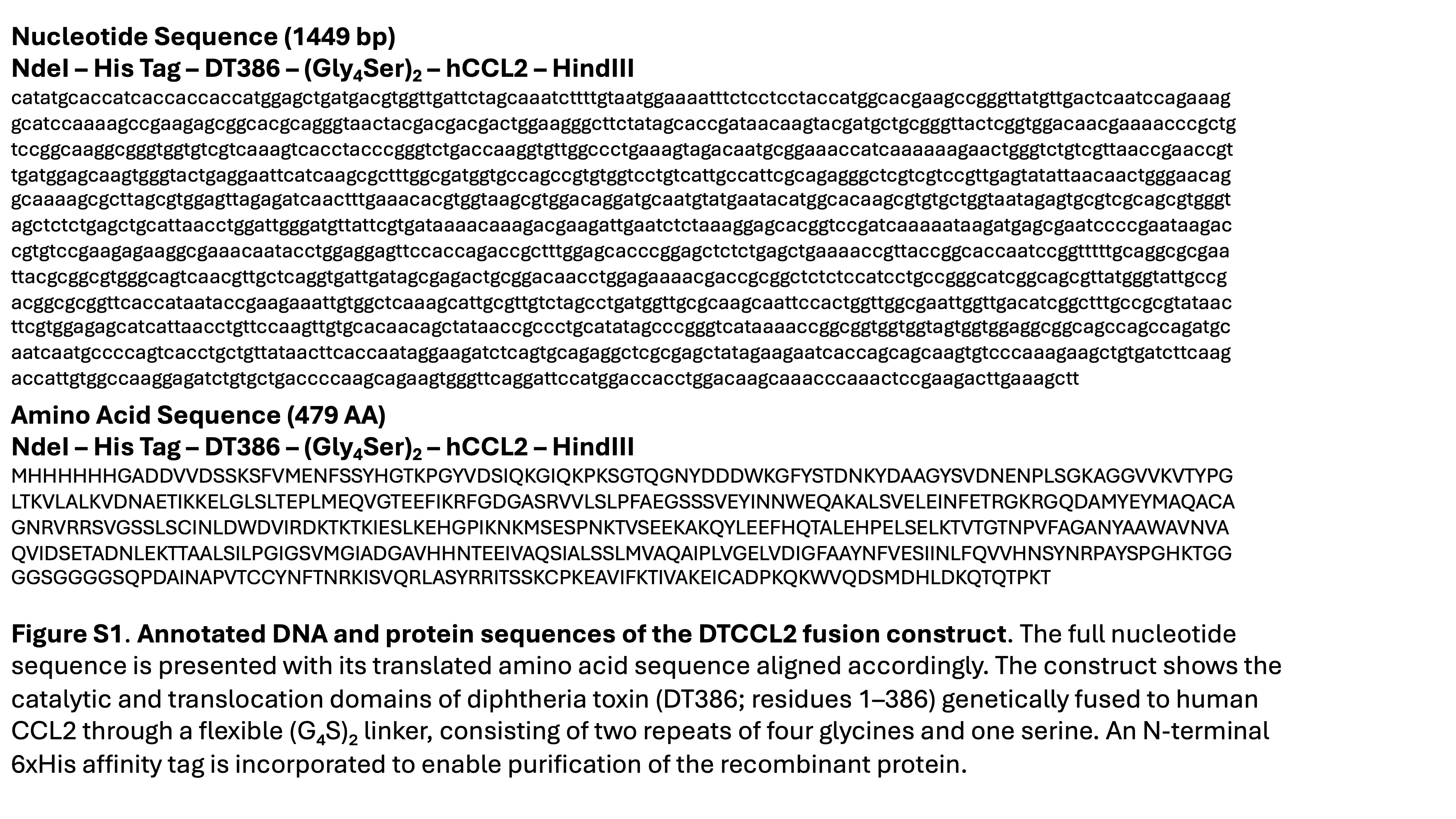

Supplement: Figure S1.jpg [file KCBT_A_2688479_SM7338.jpg]
